# Physical education across international media (2018−2024): dominant narratives and global patterns of portrayal

**DOI:** 10.3389/fspor.2026.1928709

**Published:** 2026-09-01

**Authors:** Brendon Hyndman, Vaughan Cruickshank, John Williams, Jessica Amy Sears, Ben Piggott, Brendan SueSee, Lucia Zundans-Fraser, Stephen Harvey

**Affiliations:** 1 School of Education, Charles Sturt University, Albury-Wodonga, NSW, Australia; 2 School of Education, University of Tasmania, Hobart, TAS, Australia; 3 Department of Sociology, University of Leicester, Leicester, United Kingdom; 4 School of Education, University of Notre Dame, Fremantle, WA, Australia; 5 School of Education, University of Southern Queensland, Springfield, QLD, Australia; 6 Patton College of Education, Ohio University, Athens, OH, United States

**Keywords:** curriculum, leximancer, media discourse, media representation, news media, physical education, public pedagogy, text mining

## Abstract

**Background:**

Public understandings of physical education (PE) are influenced by how the subject is portrayed in the media, yet recent international representations remain under-examined.

**Methods:**

We systematically identified English-language media articles indexed by Google News between April 2018 and November 2024. After applying pre-specified inclusion criteria, 122 articles representing 30 national or territorial focus contexts, including three transnational items, were retained. Concept patterns were identified using Leximancer as a fixed-corpus semantic mapping tool, and eight scholars conducted a three-phase interpretive synthesis involving independent review, elaboration, and consensus.

**Results:**

International media coverage most frequently connected PE with schools, physical activity, sport, fitness, students, and teachers. Physical, sporting, and institutional themes were highly connected across the corpus. Educative concepts relating to learning, development, experience, and pedagogical practice were present but were less prominent and less centrally connected.

**Conclusions:**

International media coverage predominantly represented PE through physical, institutional, and sport-oriented language, while its broader educative purposes received less consistent attention. The study provides a descriptive baseline for PE organisations, educators, and researchers considering how the subject is publicly communicated.

## Introduction

The essence of physical education (PE) has become increasingly convoluted and contested over recent decades. Scholars have described how changing political and educational agendas have contributed to conceptual confusion surrounding PE (1–4). Across this literature, curricular integration, marginalisation, and sportification have been identified as important discourses shaping how PE is positioned within schools, communities, and policy frameworks (1–4).

One line of debate has positioned PE as being exclusively concerned with the body (5). In other contexts, PE has merged with health education, creating hybrid curricular forms that sometimes dilute its disciplinary identity (6, 7). Alongside these structural changes, new terminologies have emerged, including movement literacy, physical literacy, sports literacy, and games literacy (8, 9). These literacy-based framings attempt to reassert PE's educational legitimacy, but they also contribute to conceptual ambiguity.

Curricular tensions are also evident across schooling levels. In many cases, there is a disconnection between lower secondary syllabi, which emphasise practical, performance-oriented learning, and senior schooling, which privileges theoretical knowledge about PE (3). These shifts raise questions about the coherence of PE's educational purpose and its alignment with broader curricular objectives.

In many countries, PE is not included in standardised testing regimes, which can send the message that it is less important than other curriculum areas deemed worthy of formal assessment. However, some jurisdictions (for example, in the United States) have implemented fitness or physical activity testing (10), illustrating variability in how PE is positioned within accountability frameworks. As a result, PE is often one of the first curriculum areas to face reductions during budgetary constraints (11, 12). Marginalisation has also been linked to staffing difficulties, with PE frequently undervalued in educational policy and practice (7). In many primary schools, access to a specialist PE teacher is limited, further undermining the subject's quality and legitimacy (7).

The consequences of marginalisation are significant for students because underfunding, limited specialist provision, and inadequate staffing can reduce the quality and consistency of PE provision (7, 11, 12). These systemic decisions concerning assessment, resourcing, staffing, and accountability can further erode PE's standing within schools.

Another discourse shaping PE is sportification. Historically, PE has been strongly influenced by sporting, military, and scientific ideologies that have emphasised sexism, mesomorphism, individualism, healthism, and elitism (13). These legacies continue to influence how PE is structured and perceived. In many contexts, PE has been positioned as a vehicle for elite sport development, serving to train competitive athletes and elevate the status of the profession through association with sporting success (2, 14). This emphasis has narrowed PE's pedagogical scope, often privileging competitive performance over inclusive, educative experiences.

In recent years, outsourcing PE instruction to sporting organisations has become increasingly common, particularly in primary or elementary schools (15). While outsourcing may provide access to specialised skills, it can also entrench a sport-focused discourse that insufficiently differentiates content to align with curricular standards and broader educational frameworks (15). With so many competing agendas, stakeholders, and interests, maintaining a coherent and credible PE identity within schools remains a persistent challenge (16, 17). These disciplinary debates provide an important foundation for the present study because media coverage can reproduce, simplify, or challenge the same curricular, marginalisation, and sportification discourses through which PE is publicly understood (17, 19).

Media influence is therefore an important dimension of how PE is conceptualised and valued beyond the profession itself. Weber and Monge (18) noted the challenges that have arisen in education with the transition from print-based to digital and online media. Jung (17) argued that the multitude of discourses surrounding PE can be amplified through media channels, influencing how the profession is perceived. Until recently, however, the role of media in constructing and disseminating PE narratives to the public and policymakers has been largely overlooked (19).

Despite the limited attention to PE specifically, there has been sustained interest in the broader influence of media on education. Baroutsis and Lingard (20) and Hookway and Cruickshank (21) described how mass media can shape public opinion and policy debates. Research in this area highlights the emergence of ‘public pedagogies’, where children and young people learn through immersion in media content outside formal classrooms (22). This influence can be problematic when cinematic portrayals depict PE classes as sites of bullying or exclusion, reinforcing negative stereotypes (23).

In one of the few international studies of PE media, Hyndman et al. (19) identified a restricted focus on the physical dimension, with themes centred on requirements, reporting, and law, as well as ‘exercise is medicine’ discourses linking PE to the obesity crisis and health promotion. While this work demonstrated the potential of media analysis to reveal dominant narratives, it also highlighted the need for updated and more comprehensive research in the current media environment.

Educational discourses in the media are often crisis-oriented, emphasising deficits or negative framings of teaching and learning (24, 25). Media can act as a starting point for public debate, and its ability to frame narratives shapes how education is valued. For instance, governments are influenced by how media messaging is presented, with both positive and negative implications for policy (26). The global reach of social media platforms has further amplified this influence, providing constant exposure and rapid dissemination of information (27). With its capacity to sway mass audiences (28), the media wields particular influence over marginalised subjects such as PE (7, 11).

Media reporting is also one of the most powerful forces in shaping public viewpoints about local and national policies (24). As a ‘fourth estate’, media serves as a critical actor in constructing the knowledge base from which both public authorities and the wider community understand PE (28). Consequently, the profession's identity, value, and role in education are continuously negotiated through media discourses.

Media representations can influence public perceptions, educational reforms, and curriculum decisions (29, 30). Through framing and headline selection, media coverage can legitimise or challenge particular education policies and reforms. For PE, this dynamic is particularly significant because the subject is already marginalised and vulnerable to reductive portrayals (7, 11). When the media emphasises PE as primarily about sport or health, such framings can narrow policy agendas and obscure broader educative aims. Therefore, analysing how PE is represented in international media provides critical insight into the forces that can either constrain or expand the subject's role in policy and practice.

Despite recognition that media strongly influences public opinion and education policy, limited systematic research has examined how PE is represented across international media platforms in recent years. Where studies have been conducted, they have primarily focused on narrow timeframes or specific regions (19).

This study addresses this gap by analysing English-language international media coverage of PE between 2018 and 2024. Using Leximancer text mining and structured interpretive synthesis, the study aimed to identify and interpret the dominant and less prominent themes, concepts, and discursive patterns through which PE was represented. It also examined how the prominence of these patterns compared with the earlier five-year international media analysis of PE (19).

## Materials and methods

### Study design and approach

We conducted a mixed computational text-mining and interpretive analysis of international media reporting about PE. Leximancer was used to identify concepts and themes algorithmically; findings were then interpreted through a structured, reflexive synthesis by eight scholars with expertise in HPE/education. Leximancer analyses the content of text-based documents to generate overviews of lexicographic data, identifying relationships between words and concepts (31). It produces a ranked list of the most relevant concepts based on word frequency, context, and proximity (32). By applying a systematic algorithm, Leximancer reduces potential bias associated with manual coding by ensuring that all concepts are identified consistently across the dataset. For example, variations such as ’student’ and ’students’ are automatically clustered into a single concept, providing consistency and reducing the risk of researcher preconceptions shaping early analysis.

Leximancer was used as a systematic semantic mapping tool to support transparent, whole-corpus analysis of the media dataset. Its value for this study lies in applying the same concept-identification process across all retained articles, thereby reducing the risk that early researcher assumptions or selective reading would determine the dominant themes (31). The software identified concepts and relationships through patterns of word frequency, proximity, and co-occurrence within the fixed corpus, producing ranked concepts, theme clusters, and visual maps that could be revisited and checked against the original texts. These outputs were not treated as interpretations in themselves. Rather, they provided a structured analytical starting point for researcher-led interpretation, comparison, and contextualisation. This approach enabled the study to combine computational consistency with interpretive depth, while maintaining a clear audit trail between the retained media articles, the automated outputs, and the final thematic claims.

**Figure 1:**
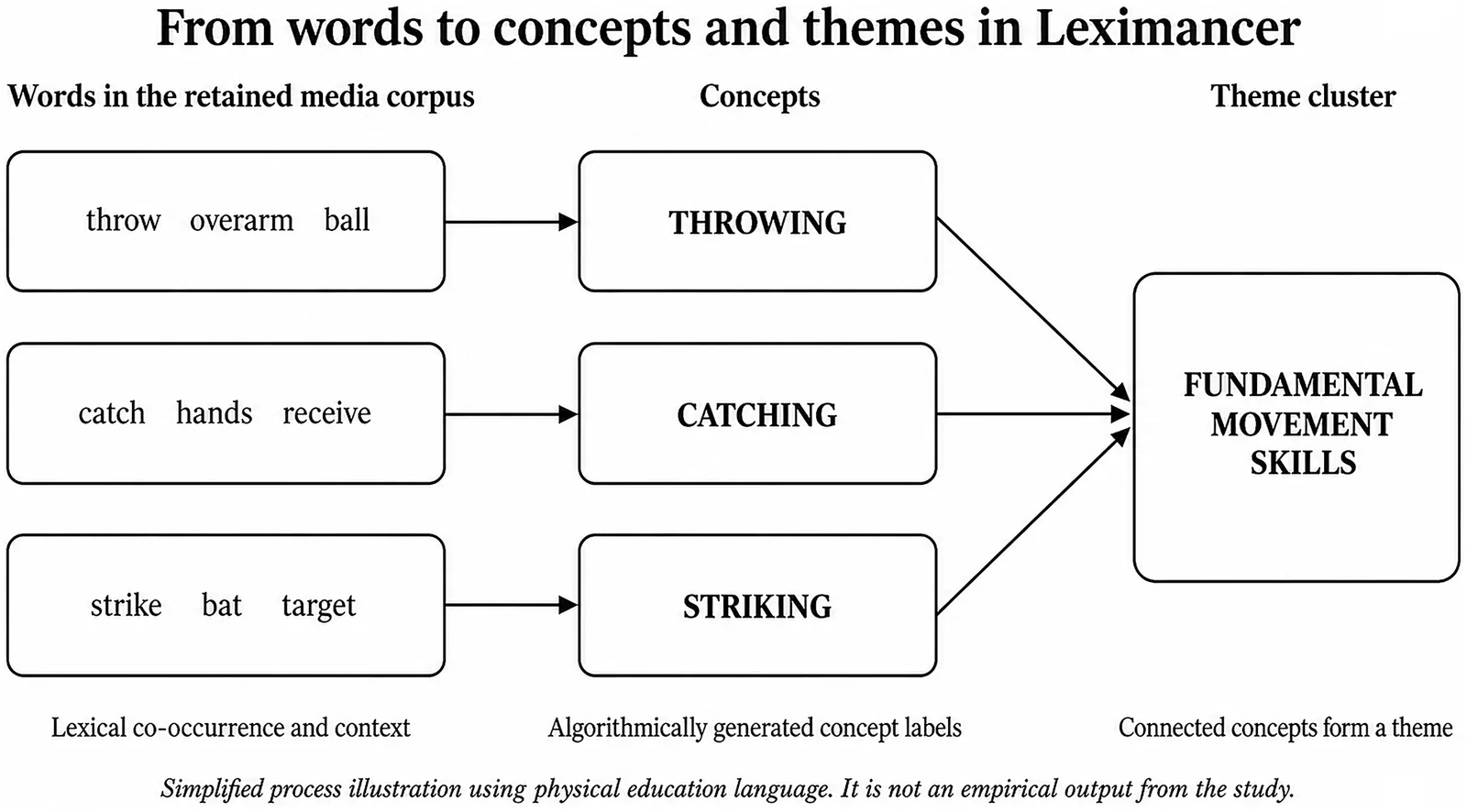
PE-specific illustration of the Leximancer pathway from words to concepts and themes. The schematic demonstrates how related words may be grouped into concepts and broader themes. It is an explanatory example rather than an empirical output from the study corpus.

The software’s process of refining text into words, concepts (clusters of words), and themes (clusters of concepts) is illustrated in Figure 1.

Leximancer has been widely applied across business, accounting, and education (32), though it remains underutilised in PE research. Existing applications have included studies of overcoming negative past experiences (33), analysing syllabi, investigating professional use of social media (34), and examining leadership practices (35). Building on these developments, the present study applied Leximancer to investigate how PE is represented in international media.

From an ontological perspective, this study adopts a relativist stance that multiple socially constructed realities coexist within media representations of physical education. Each article reflects particular cultural, political, and institutional logics rather than an objective truth. Epistemologically, the study aligns with interpretivist constructivism, where meaning is co-produced between text and researcher (24, 36). Leximancer was not used to uncover a single reality but as a heuristic device to trace discursive patterns that could then be interpreted through scholarly dialogue and contextual expertise. This positioning recognises that data are not neutral and that interpretation is inherently shaped by the researchers’ disciplinary orientations and prior experiences.

### Search strategy and information sources

A structured search of Google News was undertaken in December 2024 using the exact phrase ‘physical education’, with no geographic filter and English-language restrictions applied. Google News was selected because it aggregates media content across international outlets and enabled direct methodological comparison with the earlier international study that used the same platform (19, 63). The exact phrase was selected to prioritise coverage explicitly labelled as physical education and to reduce retrieval of broader sport, exercise, fitness, or physical activity items without an explicit PE connection. Additional synonyms were not used because the study specifically examined media representations explicitly labelled as physical education.

Searches were conducted using the platform's default relevance-based ranking, which algorithmically curates results based on factors including recency, source prominence, and user-independent relevance signals. The sampling window spanned April 2018 to November 2024, enabling analysis across pre-pandemic, pandemic, and post-pandemic periods while also increasing yield in response to the relative scarcity of PE-specific media reporting. This period was selected to extend the previous five-year international media analysis of PE and to provide a bounded account of media portrayals during a period of substantial disruption and recalibration in schooling, health, physical activity, and digital media communication. Given the dynamic and continuously updating nature of Google News, the corpus represents a time-bounded and platform-contingent sample of retrievable English-language digital news coverage. This approach enables systematic identification of dominant media discourses while acknowledging that results reflect the structure and ranking logic of the platform at the time of retrieval.

**Figure 2:**
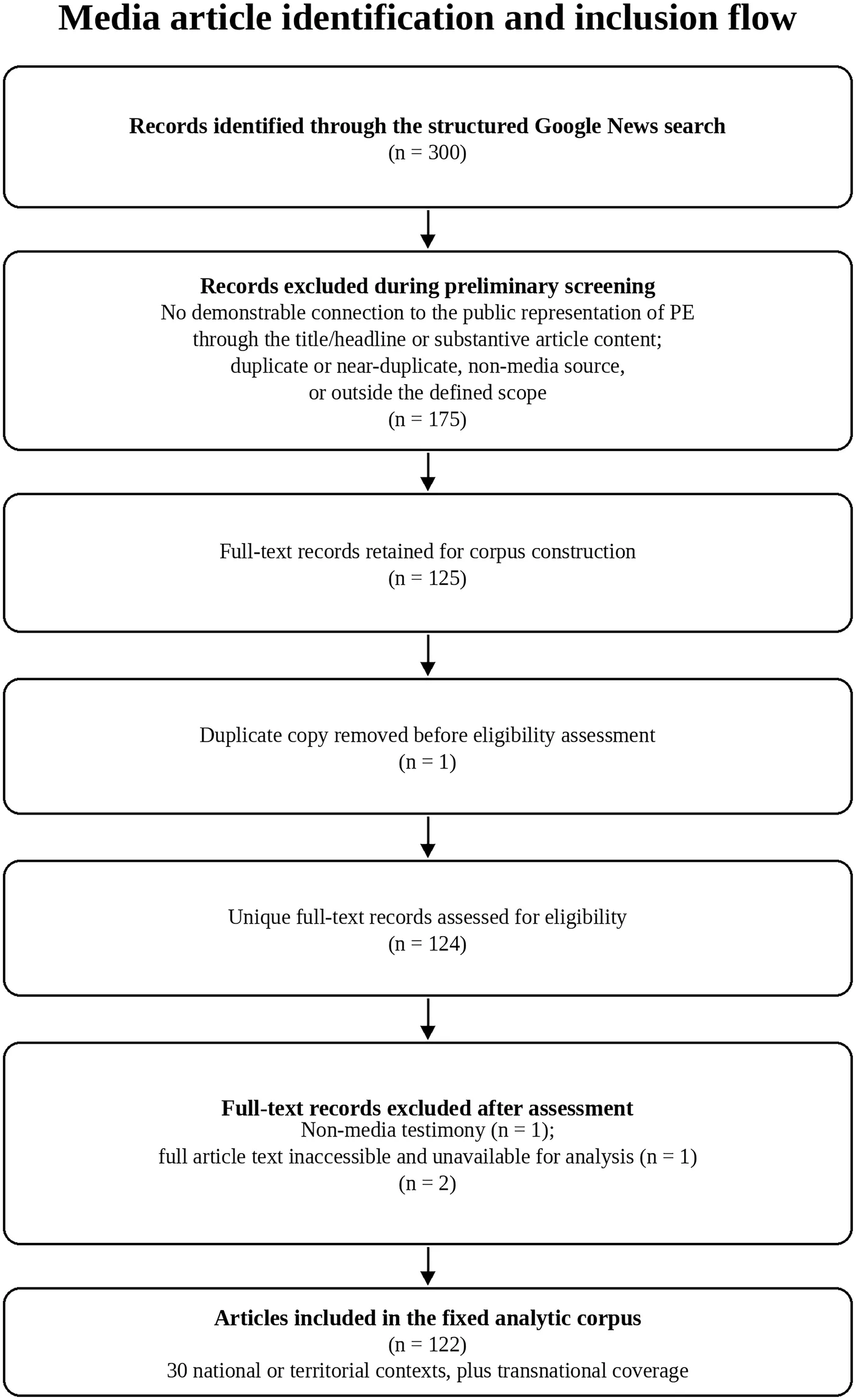
Study-specific flow diagram showing the identification, screening, eligibility assessment, and inclusion of media articles in the analysis.

The methodological approach built on a previously published international media analysis of PE that also used Google News and Leximancer to examine dominant discourses in English-language media coverage across an earlier time period (19). The present study extended that work by applying a closely aligned platform-based search and semantic mapping approach to a new temporal window, while also incorporating a more explicit interpretive synthesis and transparency measures in response to contemporary reporting expectations. The study-specific media article identification, screening, retrieval, and inclusion process is summarised in Figure 2.

#### Eligibility criteria

Articles were included if they (i) explicitly focused on PE through the title, headline, or dominant article thread; (ii) had a demonstrable connection to the public representation of PE, rather than referring only to general sport coaching, physical activity, health, exercise, or fitness without an explicit PE connection; (iii) were accessible online at the time of collection; and (iv) were written in English. Opinion and commentary pieces were assessed using the same eligibility criteria. Duplicates, syndicated repeats, and articles where ‘PE’ meant something other than physical education were excluded. Because the study examined public representation, articles in which the PE teacher identity was central to professional or legal coverage were eligible as representations of the profession. Such records were not interpreted as evidence of PE curriculum, pedagogy, teaching quality, or the conduct of PE generally.

The retained corpus included conventional news reports, institutional or organisational news items, and opinion or commentary pieces indexed by Google News. Source types were classified for descriptive reporting in Table 2, and all items were assessed using the same eligibility criteria. Accordingly, the terms media articles and media coverage are used when referring to the complete corpus, while news reports refers specifically to items classified as conventional news reporting.

### Selection process

An initial pilot screen of 20 items refined the operationalisation of criteria. One researcher screened all records; uncertain cases were checked by a second researcher, with discrepancies resolved via discussion. Where multiple versions of the same article appeared, including syndicated or republished content, only one instance was retained. In such cases, the earliest complete or most information-rich version was selected. Final inclusion yielded 122 unique articles across 30 national or territorial contexts, plus three transnational items (7−37 per corpus year). Corpus year and month reproduce the original study filing and sampling allocation, whereas publication date records the date displayed by the publisher where available. Any difference is reported transparently in Supplementary Material S1. Although search algorithms may privilege English-speaking and Western sources, the research team acknowledged this limitation as part of the interpretive scope rather than a methodological flaw, consistent with interpretivist approaches emphasising transparency of boundaries and assumptions (37). Consistent with the platform-based corpus design, transparency is supported through explicit reporting of eligibility criteria, screening procedures, duplicate-handling rules, and the final source register. The final corpus and screening audit are provided in Supplementary Material S1.

### Data preparation

Prior to analysis, article texts were cleaned to remove non-discursive elements including navigation menus, advertisements, hyperlinks, and embedded media. Headlines and main article bodies were retained. Texts were exported to.txt format and imported into Leximancer (v5). Selected manual concept merges were applied conservatively to improve semantic coherence (e.g., physical activity/PA; student/students), focusing on obvious lexical variants and abbreviations.

### Text-mining analyses

Leximancer automatically identified concepts based on frequency, proximity, and co-occurrence, generating visual maps of concept clusters and their relationships. The analysis was conducted on the cleaned, fixed corpus of retained media articles, which meant that concepts and themes were generated from the same bounded dataset used for interpretive synthesis. The ranked concepts, theme maps, and representative text segments were used to identify recurring semantic patterns across the corpus. Manual concept merges were limited to obvious lexical variants and abbreviations to preserve the integrity of the automated mapping process. Leximancer outputs were then checked against source texts during interpretation to ensure that thematic claims remained grounded in the retained articles.

#### Interpretive synthesis

To contextualise the automated outputs, the eight members of the author team undertook a structured interpretive synthesis. They were included because of their expertise in HPE or education and their role in interpreting the study's PE scholarship, policy contexts, and source texts. Selection was based on relevant disciplinary and analytical expertise rather than age or gender as sampling variables. The analysts were authors rather than research participants, and personal demographic characteristics were not analysed as study data. The interpretive synthesis was undertaken between December 2024 and April 2025. The unit of interpretation was the relationship between Leximancer-generated themes, concept rankings, and the underlying corpus. In the first phase, each scholar independently reviewed the theme maps, concept rankings, and representative text segments to identify dominant, marginal, and absent discourses. Dominant discourses were identified through their prominence in the outputs, including relative frequency, centrality within the maps, and recurrence across the corpus, while marginal discourses were identified as those that were weakly connected or comparatively infrequent. In the second phase, scholars elaborated these initial interpretations by situating the outputs within relevant HPE scholarship and policy contexts, including comparison with the earlier five-year international media analysis of PE (19). In the third phase, interpretations were compared, challenged, and refined through iterative discussion and repeated consultation of the source texts until agreement was reached. Where divergent interpretations emerged, these were explored explicitly and resolved through reference to both the automated outputs and the original articles. Analytical robustness was strengthened by retaining a clear distinction between automated pattern identification and scholarly interpretation. Leximancer provided a consistent map of recurring semantic relationships across the corpus, while the research team examined the significance of those relationships in light of PE scholarship, policy contexts, and the source texts themselves. Interpretations were repeatedly checked against the concept rankings, map structures, representative extracts, and original articles. This process helped ensure that claims were not based on isolated examples or researcher preference, but on patterns visible across the corpus and then subjected to collective scholarly scrutiny. This process constituted analyst triangulation and strengthened the credibility, dependability, and confirmability of the findings (38, 39). Because the study did not involve manual coding of individual text segments, formal inter-rater reliability statistics were not applicable. Instead, analytical rigour was supported through analyst triangulation, iterative consensus processes, and repeated cross-checking against the source corpus.

### Researcher positionality and reflexivity

Authors are HPE scholars/teacher educators (Australia, United Kingdom & United States). We acknowledge a disciplinary orientation that values educative purposes of PE, which may sensitise interpretations toward pedagogical/marginalisation discourses. Reflexive memos and group challenge were used to surface assumptions; interpretive claims were grounded in corpus evidence and cross-checked against automated outputs.

### Ethics

This study analysed publicly accessible media articles identified through a structured search of the Google News platform and did not involve human participants or identifiable private personal data; ethics review was not required under institutional policy.

### Theoretical underpinning

Interpretivist theory (40) underpinned this study, emphasising the role of researchers in constructing meaning from data through their professional knowledge and lived experiences (41). Interpretivism aims to understand, explain, and demystify social phenomena, and is particularly relevant in qualitative research where insights are shaped inductively (36).

The use of Leximancer aligns with interpretivist principles by providing systematic identification of concepts and themes while leaving scope for researchers to interpret their significance. The software reduces the risk of selective early theme generation by mapping semantic relationships across the entire dataset (32), yet the outputs do not provide meaning in themselves. This distinction is important because Leximancer generates structural maps of potential concepts, but interpretation and meaning-making rest with the researchers. As Maher (39) argued, algorithmic tools can be integrated within interpretivist research when outputs are understood as organising devices that support, rather than replace, human interpretation. Meaning is derived when researchers apply contextual expertise to evaluate why certain discourses appear, how others are absent, and what these patterns reveal about PE's representation in media.

To ensure conceptual coherence, the study’s ontological, epistemological, and methodological assumptions were aligned from the outset, as summarised below in Table 1.

**Table 1:** Research dimension alignments with study positioning.

Research dimension	Study position
Ontology	Relativist. Assumes multiple socially constructed realities embedded within media discourses of physical education.
Epistemology	Interpretivist constructivism. Knowledge is co-created through researcher engagement with the textual data and contextual expertise.
Methodology	Qualitative text-mining and interpretive synthesis. Integrates algorithmic consistency (Leximancer) with reflexive, triangulated interpretation by researchers.

Source. Authors’ methodological synthesis.

This alignment ensures that the interpretivist paradigm guides every phase of the research, from data collection through to interpretation and discussion.

By situating the Leximancer outputs within an interpretivist framework, the study acknowledges both the rigour of automated text mining and the indispensable role of human interpretation. This theoretical orientation highlights that the credibility of the findings rests not only on algorithmic analysis but also on scholars’ capacity to interpret discourses in light of their disciplinary and professional expertise. In this way, the study maintains coherence between its interpretivist paradigm and the methodological tools employed.

## Results

### Corpus profile

The final corpus comprised 122 media articles allocated to the April 2018 to November 2024 sampling window (Table 2). Corpus-year counts were 30 in 2018, 37 in 2019, 7 in 2020, 15 in 2021, 11 in 2022, 10 in 2023 and 12 in 2024. The largest focus contexts were the United States (*n* = 52), Australia (*n* = 18), India (*n* = 10) and the United Kingdom (*n* = 8). In total, the corpus represented 30 national or territorial focus contexts plus three transnational items associated with UNESCO or FISU. Outlet country and article focus were recorded separately when they differed.

**Table 2:** Corpus profile by corpus year, pandemic period and source type.

Profile dimension	Category	Articles, n	Percentage
Corpus year	2018	30	24.6
Corpus year	2019	37	30.3
Corpus year	2020	7	5.7
Corpus year	2021	15	12.3
Corpus year	2022	11	9.0
Corpus year	2023	10	8.2
Corpus year	2024	12	9.8
Pandemic period	Pre-pandemic, Apr 2018-Feb 2020	69	56.6
Pandemic period	Pandemic disruption, Mar 2020-Dec 2021	20	16.4
Pandemic period	Later period, Jan 2022-Nov 2024	33	27.0
Source type	Institutional news	59	48.4
Source type	News report	36	29.5
Source type	Opinion or commentary	17	13.9
Source type	Feature	9	7.4
Source type	Other eligible media type	1	0.8

Source. Authors’ presentation of the retained corpus profile.

For descriptive context, 69 articles were allocated to the pre-pandemic period, 20 to the pandemic-disruption period and 33 to the later period. These counts describe the temporal composition of the corpus according to the original corpus-year allocation. They do not demonstrate semantic change because Leximancer was run on the complete fixed corpus rather than separately by period.

The corpus was dispersed across 99 outlets. The Conversation contributed five articles; South China Morning Post, Monash Education and Teacher Magazine contributed four each; GOV.UK contributed three; and all other outlets contributed one or two. Institutional news was the largest source category (*n* = 59, 48.4%), followed by news reports (*n* = 36, 29.5%), opinion or commentary (*n* = 17, 13.9%), features (*n* = 9, 7.4%) and one eligible audio-feature transcript (0.8%). The prevalence of institutional sources is important when interpreting the strong presence of school, teacher, university and policy language.

The six most frequent concepts were schools (843 occurrences), physical (818), students (723), sports (634), teachers (515), and PE (494). Development ranked seventh (365), followed by need (336), classes (322), teach (274), movement (216), and activities (214). Learning ranked 15th (160), while experience ranked 18th (107). This ordering shows that educative language was present, but the strongest recurring vocabulary concerned schools, physical activity, students, sport, teachers, and PE. Table 3 presents the complete ranked list of 32 concepts. Occurrence values are Leximancer outputs. The quotations illustrate how each concept appeared in the retained corpus and are not treated as separate quantitative evidence. Every quotation is identified by Record ID, outlet, and year, with full article details provided in Supplementary Material S1.

**Table 3:** Ranked leximancer concepts and traceable illustrative quotations across the 122-article corpus.

Rank	Concept	Occurrences	Illustrative quotation	Source
1	Schools	843	“Grace is taking that class outside of school, and on her own time. She is getting school credit for virtual PE.”	PEM-2020-004; VOA Learning English, 2020
2	Physical	818	“PE is still championed for its potential to promote health and encourage lifelong physical activity… important given that over 30 per cent of pupils are ‘overweight’ or ‘obese’…”	PEM-2018-029; The Conversation, 2018
3	Students	723	“the HPE domain leader…says she can see a real change in her students…Do you have a mechanism for knowing how the fitness testing process is making your students feel?”	PEM-2020-005; Monash Education, 2020
4	Sports	634	“Brand new guidance published today will help inspire the next generation of British athletes by supporting schools to boost high-quality PE and school sport.”	PEM-2024-005; GOV.UK, 2024
5	Teachers	515	“This is where a portion of the government's funding could be of great benefit. It could help enhance teaching practices in PE, possibly by the introduction of online teacher training platforms.”	PEM-2023-006; University of South Carolina, 2020
6	PE	494	“Have a broad and balanced HPE program that goes beyond sport. Sport is a vital and importance component of any program but, if we are led by the Curriculum, ‘Games and Sports’ is only one focus area of 12 in HPE.”	PEM-2021-014; Teacher Magazine, 2021
7	Development	365	“You have the opportunity to start to develop an inventory of skills, assets and resources the young person has exemplified, or can work towards.”	PEM-2018-028; Monash Education, 2018
8	Need	336	“majority of kids need to spend more time being active both in school and at home. Additional time spent in PE increases students’ ability to learn the skills to stay active.”	PEM-2023-007; Edge Hill University, 2023
9	Classes	322	“As long as we were fostering a passion for movement, the goals of my PE class were being met. Until last year.”	PEM-2018-016; Salon.com, 2018
10	Teach	274	“It's not just come and show up…you’ve got to demonstrate that you can teach this content, demonstrate your knowledge of the subject matter and write lesson plans for that content.”	PEM-2024-002; Boise State University College of Health Sciences, 2024
11	Movement	216	“There is a real opportunity for creativity, for curiosity and for movement for movement's sake. It means exploring things in-movement, not just the tactical or technical side of things.”	PEM-2019-016; Monash Education, 2019
12	Activities	214	“setting content and activities for students to do individually and maybe even in groups, and then potentially having those scheduled Zoom sessions.”	PEM-2021-011; Teacher Magazine podcast, 2021
13	Participate	199	“state laws mandating P.E. participation have seen a sharp decline by school grade level…only 15%, 9% and 6% of students in elementary, middle and high schools in the U.S., respectively, are required to take P.E. classes on three or more days a week.”	PEM-2021-010; Washington University in St. Louis, 2021
14	Worked	177	“We want to work with partners to change the health of this state.”	PEM-2020-003; West Virginia University, 2020
15	Learning	160	“A portfolio of work is an excellent way for young people to demonstrate the knowledge, skills and understanding they’ve developed in your class. It documents a range of learning and gives an educative focus to what you do.”	PEM-2018-028; Monash Education, 2018
16	People	145	“Schools should think about changing facilities that let young people change more privately… also offer a choice of kit or let pupils wear a t-shirt over their swimming costume.”	PEM-2019-008; The Conversation, 2019
17	Fitness	130	“The fitness industry is booming, so why are PE classes disappearing?”	PEM-2019-018; The Washington Post, 2019
18	Experience	107	“Getting comfort from this experience is one of the first steps to valuing movement. It brings courage.”	PEM-2019-016; Monash Education, 2019
19	Take	100	“We need to sharpen the pencil a little bit, take stock of our impact and recognise what we are doing has value. What do they bring to the table? It's a nice approach to take in your teaching.”	PEM-2018-028; Monash Education, 2018
20	Student	90	“After winning on Italian soil, the student has new plans. “I’m very happy I’ve won this first international competition.”	PEM-2018-013; PUCRS English website, 2018
21	Kids	89	“Everyone agrees that preventive medicine has value, but it's a longer investment, and we know behavior change can be difficult…Focusing on kids makes complete sense.”	PEM-2020-003; West Virginia University, 2020
22	Requirement	77	“In the 1900s, virtually all U.S. colleges had a physical education requirement. In 1942…Gates announced the end of required Physical Education.”	PEM-2018-027; The Daily Pennsylvanian, 2018
23	Exercise	76	“When these kids get into marching band, unlike a semester of PE, they end up staying three, four years. So they’re getting the exercise over that longer period of time.”	PEM-2018-005; WCNC, 2018
24	Team	73	“It's a starting point. You can build on those strengths and resources to support each other so that, collectively, the team progresses.”	PEM-2018-028; Monash Education, 2018
25	Day	64	“I agreed to sit down with her and ask how she felt about it. It was an illuminating conversation, taking place on a sunny fall day at recess.”	PEM-2018-016; Salon.com, 2018
26	Become	60	“their confidence grows. They also become more willing to go beyond their normal comfort levels and overcome their fears.”	PEM-2018-004; K-12 Dive, 2018
27	Things	51	“The words we use matter and how we perceive things matter. How we frame things makes a difference. We are ‘on’ all the time. We are trying new things.”	PEM-2018-016; Salon.com, 2018
28	Minutes	51	“the State Senator wants to change that to 150 min (of PE) a week, no matter what.”	PEM-2018-020; WQAD.com, 2018
29	Challenges	51	“The change process a came with a number of challenges that needed to be addressed. The lack of resources was a primary challenge, so innovation was the name of game.”	PEM-2019-013; Jamaica Gleaner, 2019
30	Grades	42	“Depending on their grade, students may have a soccer ball, weights, resistance bands, jump rope, beanbags, or paddles for pickle ball in their bags.”	PEM-2021-004; California Teachers Association, 2021
31	Family	34	“We had parent involvement because we recognize the importance of the family. Kids are not the ones going to the grocery store.”	PEM-2024-013; UNM Newsroom, 2024
32	University	32	“Admission quotas and subsequent years and the amount of tuition will be determined by the university in coordination with the Ministry of Physical Education.”	PEM-2019-033; Kun.uz, 2019

Source. Authors’ presentation of Leximancer v5 outputs from the retained 122-article corpus. Illustrative quotations are drawn from the retained corpus, with full article details in Supplementary Material S1.

### Concept-map relationships

The authentic Leximancer map showed dense relationships among the high-frequency concepts schools, physical, students, sports, teachers and PE. In this context, proximity and connectivity indicate that concepts repeatedly occurred near one another across the corpus. They do not establish causation, journalistic intention or a single evaluative meaning. The map therefore supports a descriptive conclusion that media portrayals commonly connected PE with school settings, bodily activity, sport, students and professional actors.

Educative concepts were not absent. Development, teach, movement, learning and experience appeared within the ranked output and source quotations. Their comparatively lower frequency or weaker integration, however, suggests that detailed accounts of curriculum learning and pedagogical processes were less consistently foregrounded across the corpus than institutional, bodily and sport-related language.

**Figure 3:**
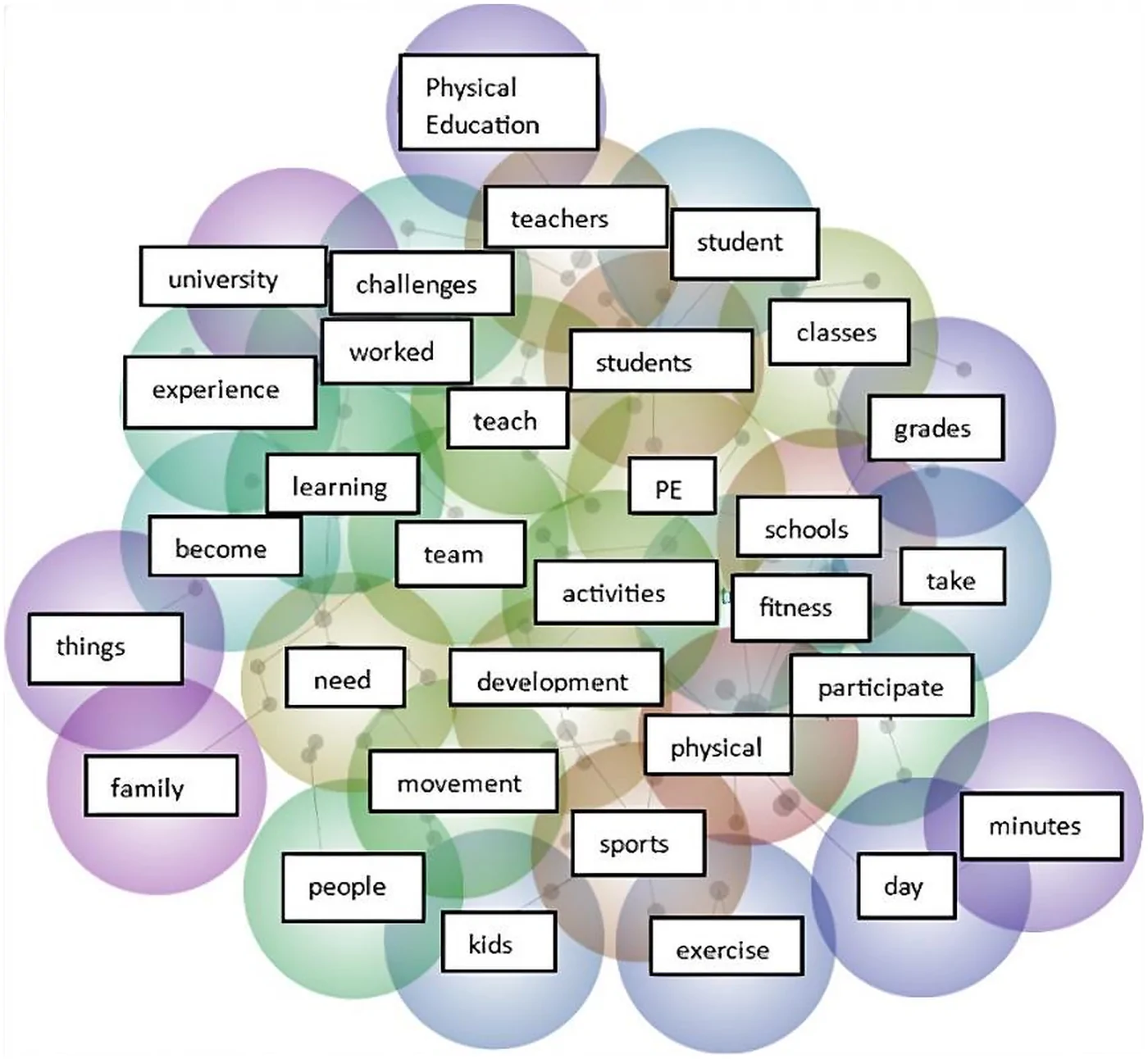
Authentic Leximancer concept map for the fixed corpus of 122 media articles. Warmer colours indicate greater thematic connectivity within the software output. Spatial proximity represents recurring co-occurrence relationships and should not be interpreted as causation or evaluative direction.

Within the concept map, ‘teachers’ and ‘teach’ were prominent, while connections with ‘curriculum’, ‘assessment’, and ‘professional learning’ were less visible (Figure 3). Descriptively, teacher-related reporting appeared more frequently alongside delivery and activity language than explicitly pedagogical language. This pattern should not be interpreted as proof of how teachers are represented in all media coverage; rather, it identifies a tendency within the analysed corpus.

Although relatively infrequent, the emergence of ‘family’ and ‘university’ as identifiable themes (Table 3) warrants attention. References to ‘family’ commonly appeared in media about parental involvement and community responsibility for supporting active lifestyles, suggesting a broadening of PE discourse beyond the school gate. Mentions of ‘university’ reflected higher education's role in shaping teacher preparation, research, and public commentary on PE policy. Together these lower-frequency ranked themes hint at an expanding social ecosystem influencing how PE is understood and valued.

A small number of source quotations used phrases such as ‘movement for movement's sake’ and ‘curiosity in-movement’ (Table 3). These extracts demonstrate that aesthetic, exploratory, and embodied language was present within the corpus, although it was not sufficiently prominent to be characterised as a widespread counter-narrative.

The full corpus included media articles across 30 national or territorial contexts, plus three transnational items. Table 4 presents separate Leximancer outputs for the higher-volume country groupings used in the comparative analysis. Contexts represented by smaller numbers of articles remained in the overall corpus but were not analysed as separate subsets.

**Table 4:** Country-specific leximancer outputs for the higher-volume comparative subsets.

Other Asia-Pacific countries (*N* = 12 media articles)			Australia (*N* = 18 media articles)			India (*N* = 10 media articles)			United Kingdom (*N* = 8 media articles)			United States (*N* = 52 media articles)		
Theme^a^	Sections connected to overall theme	Automated concepts within the overall theme	Theme^a^	Sections connected to overall theme	Automated concepts within the overall theme	Theme^a^	Sections connected to overall theme	Automated concepts within the overall theme	Theme^a^	Sections connected to overall theme	Automated concepts within the overall theme	Theme^a^	Sections connected to overall theme	Automated concepts within the overall theme
Physical	64	physical, school, classes, daily	Students	362	students, teachers, school, PE, teaching, time, activities, online, primary, important	Central Board of Secondary Education (CBSE)	49	CBSE, physical education, board	PE	127	PE, schools, pupils, develop	Physical	383	physical, education, activity, health, fitness
Activity	56	activity, people, time, schools, children, hours, young, week, least	Physical	226	physical, education, health, activity, curriculum, fitness, sport	Students	42	Students, year, class	Sport	76	sport, children, active, guidance, young, important, healthy	Students	330	students, teachers
PE	32	PE, sports, exercise, lessons, opportunity	HPE	205	HPE, learning, skills, movement, opportunities, develop, support, student, provide	Schools	32	Schools, physical, period, Health, classes, HPE, compulsory	School	63	school, boys, health, week	School	295	school, class, teacher, schools, elementary
Students	44	Students, university	People	39	Teacher	Sports	17	Sports	Physical	59	Physical, support	PE	221	PE, kids, work, exercise
Teachers	29	Teachers, course, football, training	Research	29	Research	Examination paper	15	Paper, asked, questions, examiner	Gender and Sport	52	sports, girls, opportunities	Time	197	time, skills, activities, sports, active, during

Source. Country-specific analysis of Leximancer v5 outputs from the retained corpus.

The Other Asia-Pacific countries grouping comprised China (*n* = 2), Fiji (*n* = 1), Hong Kong SAR (*n* = 2), Indonesia (*n* = 1), Japan (*n* = 2), New Zealand (*n* = 2), the Philippines (*n* = 1), and Thailand (*n* = 1), totalling 12 media articles. Australia and India were analysed separately. Media articles from Guyana, Kazakhstan, Malta, Mexico, Namibia, and Uzbekistan remained in the full corpus but were not analysed as separate country subsets because of the small number of articles from each context. Themes and concepts were generated using Leximancer v5.

^a^ Theme labels were automatically generated by Leximancer v5.

Table 4 indicates that PE was framed differently across contexts. Institutional and examination language was more visible in India, sport and gender in the United Kingdom, and school, student, and physical-activity language across the larger United States and Australian subsets. These patterns should be interpreted as contextual emphases rather than direct national comparisons because the subset sizes were uneven and the Other Asia-Pacific grouping combined several jurisdictions.

## Discussion

The Discussion is organised into five parts. It first considers the overall media portrayal of PE and its public-pedagogical significance, followed by the dominant physical and sport discourses, the less prominent educative and student-centred themes, sociocultural and policy variations, and the study's strengths and limitations.

### Media portrayals of PE and public pedagogies

Analysing the portrayal of PE across international media and extending insights beyond the initial 5-year study is essential to understand trends in media communication about PE. This study fills a gap in global PE scholarship by providing long-term insights through a transparent, fixed-corpus text-mining approach combined with structured interpretive synthesis. Since the previous media reporting study (19), educational organisations, in addition to media organisations, have expanded their capacity to produce news media articles that align with Google News platform functions. For example, Springfield College in the United States and Monash University in Australia now have article-generating systems, with the concept of research even appearing in the Australian-specific reporting of PE. Although universities emerged as a theme, schools remained the most dominant context for PE media reporting, confirming that school settings continue to be the main reference point for global discussions of PE.

Since the 2013−2018 period, the world has experienced major transformations from the lockdowns and health protocols associated with the COVID-19 pandemic, particularly in relation to PE (42). Identifying the messages or ‘public pedagogies’ communicated through media to governments, stakeholders, and the broader public can reveal conflicts between how the PE profession seeks to represent itself and how it is portrayed externally (22, 23). This study addresses the research gap by identifying recurring representations of PE within the analysed English-language international media corpus. The findings show which themes were most and least prominent and provide a focused evidence base for PE organisations, educators, and researchers considering how the subject is publicly communicated. Media framing is relevant to this work because it can shape public perceptions of education and the PE profession (28). From an interpretivist standpoint, the findings represent a co-construction between the data and the researchers. The meanings identified are understood not as objective truths but as negotiated interpretations emerging from the researchers’ engagement with the text and context. Recognising this interpretive process foregrounds how public discourses both reflect and shape professional realities in PE. Because PE is often described as a ‘contested ground’ with competing discourses and unclear representations (1, 3, 4, 43), it is crucial to identify which narratives dominate public portrayals.

### Dominance of physical and sport discourses

How PE has been understood and practiced has changed little since the 1950s (44). This continuity can be attributed to power struggles within the profession and the influence of habitus, defined as individuals’ ingrained structures of thought and behaviour (45). Many stakeholders continue to privilege and reproduce discourses of ‘PE-as-sport-techniques’ (44), reinforcing the long-term dominance of sport and fitness-focused representations (46). Such discourses provide context for the strong visibility of sport and fitness in the findings. Yet contemporary scholarship has argued for more holistic understandings of PE that recognise quality provision as including cognitive, social, and affective dimensions alongside physical ones (47). Although PE is a major curriculum subject, the analysed media corpus contained limited explicit attention to assessment, professional development, teacher qualifications, social media, and staffing. This pattern suggests that educative and professional dimensions were less visible than physical, sport, and institutional framings within the corpus (7, 11).

Although PE has increasingly been linked with social-emotional pedagogical approaches, digital practices, and progressive pedagogies, little evidence of these shifts appeared in the media reporting analysed. Instead, a ‘PE for health and sport’ discourse was prominent, with major themes centring on movement, fitness, exercise, activities, sport, and physicality. Several source quotations linked PE or physical activity with health improvement, obesity-related concerns, and preventive health. These examples were consistent with the broader prominence of physical, fitness, and health-related language within the corpus. This echoes earlier concerns that PE can be framed primarily as a physical or biomedical solution rather than an educative process (48). The findings therefore identify a continuing need to examine how PE's broader educative purposes are communicated in public media (7, 11).

### Emerging educative and student-centred themes

Student-related language was prominent in the corpus. ’Students’ ranked third overall and was the second most prevalent theme in the United States subset. Within the United States reporting, this prominence appeared alongside themes concerning time, classes, teachers, and activity. These patterns can be considered alongside contemporary PE standards that emphasise social and emotional competencies, authentic movement experiences, connection, and engagement (49, 50). However, the media data do not show how students themselves interpreted these messages.

Less prominent themes including ‘development’, ‘learning’, ‘experience’, and ‘team’ provided examples of educative language beyond fitness and sport. Their lower rankings indicate that such language was present but not dominant. These themes are consistent with scholarship framing PE as supporting confidence, competence, collaboration, communication, and resilience (51–53). Taken together, the findings show a contrast between the high visibility of students as subjects of reporting and the lower visibility of explicitly educative purposes.

### Sociocultural and policy variations

Themes related to online learning and digital pedagogies were rarely observed, even though the reporting period spanned the COVID-19 pandemic. This limited attention reflects PE's broader marginalisation, since numerous studies highlighted that PE provision was significantly reduced or redefined during the pandemic (42, 54–56). In many cases, online PE was reduced to physical activity breaks rather than meaningful learning experiences. The lack of coverage in the media suggests that these pedagogical challenges received little attention in public discourse.

The results also revealed the growing prominence of the sport theme. Compared to its position as the 11th-ranked theme in the earlier five-year analysis (19), sport was ranked fourth overall in the current study. Country-specific variations highlighted how cultural and policy contexts shaped this theme. In the United Kingdom, sport and gender-related discourses reflected government efforts to ensure equitable access to PE and sport for all students, particularly girls (57). Yet sport remains a contested discourse, as competitive agendas have historically limited inclusivity and driven many girls away from PE (58). In contrast, sport was largely absent from US-specific reporting, despite sport programs receiving greater funding and visibility compared to PE (49, 59). In Australia, the reduced prominence of sport compared with the earlier five-year study (19) reflects shifts toward informal and participatory forms of physical activity (60, 61). In India, the dominance of examination-related themes such as CBSE and ‘examination paper’ reflects a high-stakes academic culture that positions PE as instrumental to credentialing and future career pathways (62). These cross-national differences illustrate how global narratives of PE are filtered through national policy agendas and sociocultural traditions.

### Strengths and limitations

A key strength of the study was the combination of whole-corpus semantic mapping, source-text checking, and collective scholarly interpretation. This provided a transparent link between the automated outputs and the interpretive claims while reducing reliance on isolated examples.

The main limitations were the English-language restriction, exclusion of video and other non-text formats, and reliance on Google News, which does not capture all media content (63). The corpus therefore provides a time-bounded and platform-contingent account of coverage from April 2018 to November 2024 rather than a representation of all global PE media. Findings are most transferable to English-language digital media indexed by Google News during the study period. Future research could compare non-English reporting, alternative platforms, and later periods.

## Conclusion

Across 122 English-language media articles spanning 30 national or territorial contexts, plus three transnational items, PE was most commonly represented through schools, physical activity, sport, fitness, students, and teachers. The Leximancer outputs showed that physical, sporting, and institutional themes were more prominent and closely connected than themes explicitly concerned with learning, development, experience, assessment, or pedagogical practice. The corpus nevertheless contained less frequent educative language concerning development, learning, movement experience, and student needs.

Compared with the earlier five-year analysis (19), schools remained central, sport was more prominent, and regulatory concepts such as requirements, reporting, and law were less visible. The country-specific analyses also demonstrated different emphases within the United States, Australia, the United Kingdom, and India. These comparisons should be interpreted within the boundaries of an English-language Google News corpus collected for the period from April 2018 to November 2024.

Overall, the findings show that international media coverage continues to present PE mainly through physical, sport, and school-based frames, while its broader educative purposes receive less consistent attention. The study provides a descriptive baseline for future research examining whether these patterns change across languages, media platforms, and later periods.

## Data Availability

The original contributions presented in the study are included in the article/Supplementary Material, further inquiries can be directed to the corresponding author.
